# White matter signatures of addiction diverge across opioids, cannabis and nicotine and predict impulsive choice in cannabis dependence

**DOI:** 10.1038/s41398-026-04491-8

**Published:** 2026-10-09

**Authors:** Richard O. Nkrumah, Lea Wetzel, Justin Böhmer, Katharina Eidenmueller, Patrick Bach, Silvia De Santis, Henrik Walter, Wolfgang H. Sommer, Gabriele Ende

**Affiliations:** 1 Department of Neuroimaging, Central Institute of Mental Health, Medical Faculty Mannheim, Heidelberg University, J5, 68159 Mannheim, Germany; 2 German Center for Mental Health (DZPG), partner site Mannheim-Heidelberg-Ulm, Mannheim, Germany; 3 Department of Addictive Behavior and Addiction Medicine, Central Institute of Mental Health, Medical Faculty Mannheim, Heidelberg University, J5, 68159 Mannheim, Germany; 4 Department of Psychiatry and Psychotherapy CCM, Charité - Universitätsmedizin Berlin, Corporate Member of Freie Universität Berlin, Humboldt-Universität zu Berlin, and Berlin Institute of Health, Berlin, Germany; 5 Institute of Medical Psychology, Charité - Universitätsmedizin Berlin, Corporate Member of Freie Universität Berlin, Humboldt-Universität zu Berlin, and Berlin Institute of Health, Berlin, Germany; 6 Instituto de Neurociencias, CSIC-UMH, Sant Joan d’Alacant, Spain; 7 Department of Psychopharmacology, Central Institute of Mental Health, Medical Faculty Mannheim, Heidelberg University, J5, 68159 Mannheim, Germany; 8 Bethany Hospital for Psychiatry, Psychosomatics and Psychotherapy, 17489 Greifswald, Germany

**Keywords:** Addiction, Human behaviour, Diagnostic markers

## Abstract

White matter (WM) microstructure is implicated in the neurobiology of substance use disorders (SUDs), yet direct comparisons across substance-dependence syndromes remain scarce. It is also unclear whether WM abnormalities relate to impulsive decision-making and substance use severity. We performed a cross-sectional, dual-centre diffusion MRI study and quantified WM microstructure using diffusion tensor imaging and neurite orientation dispersion and density imaging in 87 individuals with SUDs (48 with cannabis dependence (CUD), 16 with opioid dependence (OUD), and 23 with nicotine dependence (NUD)) and 53 nonsmoking healthy controls. Whole-brain tract-based spatial statistics and tract-specific analyses of the middle longitudinal fasciculus (MLF), a temporo-parietal tract implicated in cognitive control and valuation, were performed. OUD and CUD exhibited convergent, widespread WM abnormalities characterised by reduced fractional anisotropy and increased mean diffusivity and orientation dispersion across fronto-temporo-parietal and limbic pathways, including the superior longitudinal fasciculus, arcuate fasciculus, cingulum, and MLF. In contrast, NUD showed more circumscribed alterations observed primarily in MD and  localised mainly to anterior and callosal white matter regions. In CUD, higher MLF orientation dispersion was associated with steeper delay discounting (r = 0.41, *p* = 0.011). Moderated mediation analyses indicated that this association was indirectly driven by cannabis consumption, with a significant conditional indirect effects observed only among daily users. No significant MLF-discounting associations were observed in OUD. Our findings reveal both shared and distinct WM alterations across SUDs and identify a link between MLF microstructure and impulsive choice in cannabis dependence.

## Introduction

Substance use disorders (SUD) remain a major public health concern due to their strong association with poor mental health outcomes and neurocognitive impairments [1]. Individuals with SUD frequently exhibit disruptions across a range of cognitive and emotional domains, including executive functioning, emotional regulation, and reward processing [2, 3]. Recent studies suggests that different substances—such as opioids, cannabis, nicotine and alcohol—may be associated with distinct psychiatric risk profiles [4–6], although the overlap across mood, anxiety, and psychotic outcomes remains substantial [7]. For instance, opioid dependence (OUD) has been associated to increased risk of mood and anxiety disorders [8, 9], cannabis dependence (CUD) to heightened psychosis risk in genetically or environmentally vulnerabilities individuals [10, 11], nicotine dependence (NUD) to heightened anxiety and attentional deficits [12, 13], and alcohol to a broad spectrum of severe psychiatric and somatic outcomes [14].

Functional neuroimaging studies have consistently reported abnormalities across various SUDs [15]. These effects may show substance-related variation [16], shaped by unique pharmacokinetics, receptor profiles, and chronic exposure patterns [17]. However, despite growing evidence from functional neuroimaging, comprehensive analyses of substance-related effects on white matter (WM) and gray matter (GM) microstructural properties using structural neuroimaging techniques, such as diffusion magnetic resonance imaging (dMRI), have remained underexplored. Recent translational work has begun to address this gap, highlighting alterations in WM microstructure across both human and rodent models [18], and support for distinct microstructural patterns across substances has been provided by several meta-analyses [19, 20]. Diffusion tensor imaging (DTI) metrics, such as fractional anisotropy (FA) and mean diffusivity (MD), have been widely utilized to characterize WM abnormalities in SUD populations. Reductions in FA and elevations in MD are commonly interpreted as reflecting alterations in microstructural organization, including changes in axonal coherence, myelination, or extracellular space, although these metrics are not specific to any single underlying biological processes [18, 21, 22].

Localized WM alterations have been observed in major tracts across the whole brain, including the anterior thalamic radiation (ATR), superior longitudinal fasciculus (SLF), inferior longitudinal fasciculus (ILF), uncinate fasciculus (UF), fornix and corpus callosum (CC), with these changes frequently associated with executive, attentional and affective impairments observed in individuals with SUD [21]. Beyond these general findings, substance-specific WM alterations have also been reported with many studies in CUD and OUD populations, in particular, highlighting changes within fronto-temporo-parietal pathways. For example, cannabis use has been linked to WM microstructural deficits in the arcuate fasciculus (AF), SLF, UF and middle longitudinal fasciculus (MLF), as well as commissural tracts like the CC in CUD individuals [23, 24]. Similarly, opioids use has been linked to WM microstructural deficits in the UF, ILF, cingulum, and internal/external capsules in OUD individuals [25–27], consistent with their involvement in pain, emotion and reward regulation.

In contrast, individuals with alcohol and stimulant dependence often exhibit high variability in consumption patterns and neurotoxic effects, limiting the interpretability of cross-sectional findings under assumed pharmacological stability. For example, alcohol levels fluctuate rapidly due to fast metabolism, and although neurobiological dynamics occur across intoxication, withdrawal, and early abstinence phases, WM alterations are often widespread and already detectable after relatively short drinking periods at moderate levels of use [28]. Furthermore, recent evidence has also shown alcohol-induced damage in specific tracts such as the fimbria/fornix, leading to disrupted hippocampal-prefrontal connectivity even during early abstinence — suggesting a characteristic profile of alcohol-related WM pathology [29]. Similarly, nicotine use lacks a pharmacologically stable steady state due to its rapid clearance and fluctuating plasma levels and has been primarily associated with more focal WM alterations, particularly in fronto-limbic tracts such as the anterior corona radiata and cingulum [30]. These complexities has led recent studies to examine the effects of co-use, such as combined cannabis and nicotine consumption, which has been associated with decreased FA in the cingulum and fornix—tracts involved in memory and emotion regulation [31]. However, inconsistencies remain in the localization of WM alterations associated with each type of substance dependence, which may arise due to the limitations of DTI in resolving complex WM fiber architectures, given that up to 90% of WM regions contain crossing fibers [21, 32, 33].

Advanced dMRI techniques that provide more biologically specific measures such as neurite orientation dispersion and density imaging (NODDI) have been adopted to offer improved sensitivity in recent dMRI studies [34, 35]. NODDI provides compartment-specific metrics, such as the neurite density index (NDI), isotropic volume fraction (ISOVF) and orientation dispersion index (ODI), which allow for a more refined interpretation of WM microstructural properties by quantifying axonal packing and fiber orientation dispersion [36, 37]. Studies comparing DTI and NODDI in clinical populations have reported that NODDI is more sensitive to subtle changes in WM microstructure and aligns well with cognitive impairments, as observed in a sample with mild traumatic brain [34, 38, 39]. However, to date, NODDI has been sparsely applied in SUD populations, with only one study showing associations between cocaine use and increased ISOVF in fronto-parietal and temporo-parietal tracts [40].

While NODDI metrics provide more WM microstructural information, their behavioral relevance in SUD remains underexplored. Executive dysfunction, particularly impaired decision-making, is a hallmark of SUD and is typically assessed using a battery of cognitive tasks. Among these, delay discounting (DD)—the tendency to prefer smaller immediate rewards over larger delayed ones—has emerged as a core behavioral marker of impulsivity in addiction [41]. Neuroimaging studies link DD to frontoparietal network, particularly the dorsolateral prefrontal cortex (dlPFC), which supports cognitive control and future-oriented valuation [42, 43]. The middle longitudinal fasciculus (MLF), a key WM association tract, is increasingly recognized for its role in connecting temporal, parietal, and occipital regions, with projections extending towards the prefrontal cortex and has been implicated in higher-order executive functions [44]. Although the precise functioning of the MLF is still being explored, its relationship with the superior temporal gyrus, superior parietal lobule, and dlPFC suggests that it may support decision-making processes that require integration of sensory, memory, and cognitive control inputs. In SUD, altered MLF microstructure may be relevant to these pathways and to individual differences in impulsive decision-making involving delayed reward.

The present study has three primary objectives: (1) to identify WM microstructural alterations in individuals with cannabis, opioid, and nicotine dependence compared with non-smoking healthy controls; (2) to determine whether these changes reflect shared and distinct patterns across the three dependence groups and (3) to examine whether MLF microstructure relates to delay discounting, as assessed by the Kirby Delay Discounting Task, in cannabis and opioid dependence, with nicotine and alcohol use included as covariates. Given the MLF’s role in connecting temporal and parietal control regions with the prefrontal cortex, we hypothesized that its microstructural properties would be associated with delay discounting behavior in a substance-related manner. We further explored whether substance use severity, including both amount and frequency of use, was related to this brain-behavior relationship.

## Methods

### Participants

For this dual-center study participants were recruited from clinical settings and through community advertisements at two sites: the Central Institute of Mental Health (CIMH) in Mannheim, Germany, and the Department of Psychiatry and Psychotherapy at the Charité - Universitätsmedizin Berlin, Germany. The study included individuals meeting ICD-10 criteria for opioid, cannabis, or nicotine dependence (see details below), along with non-smoking healthy controls (HC). HC participants were excluded if any history of SUD was reported. Within each diagnostic group, current comorbid substance use disorder was an exclusion criterion, except for alcohol and tobacco, which were permitted across all groups and verified through structured clinical interviews. Written informed consent was obtained from all participants prior to participation. Inclusion criteria required participants to be between 18 and 55 years of age and free from major neurological conditions. Exclusion criteria included current comorbid psychiatric disorders and contraindications for MRI scanning. The study protocol was approved by the relevant institutional review boards at each site.

Demographic and clinical characteristics of all SUD groups and control participants are summarized in Table 1. Age differed significantly between groups and was therefore controlled for in all analysis. Although group differences in sex were not statistically significant, sex was included as a covariate based on prior literature supporting sex-related effects on white matter microstructure [45, 46]. Study sites were also controlled for due to their known associations with dMRI measures [47]. Target sample sizes for the four groups were informed by previously reported FA effect sizes in SUD diffusion MRI meta-analysis [21], together with a priori power considerations from the study planning phase, with approximately 40 participants planned per group. Final group sizes (HC = 53, NUD = 23, OUD = 16, CUD = 48), reflected this planning together with recruitment feasibility during the study period.

**Table 1:** Clinical and demographic data.

	Control *n* = 53	Nicotine dependence (NUD) *n* = 23	Opiate dependence (OUD) *n* = 16	Cannabis dependence (CUD) *n* = 48	Statistics
Age in years (SD)	33.83 (7.55)	39.04 (10.45)	41.38 (7.23)	32.85 (7.37)	*F* = 6.844 *P* = < 0.001
Sex (males %)	23 (43.40)	13 (56.52)	12 (75)	30 (62.50)	*X*^2^ = 6.573 *P* = 0.087
Site (Berlin/Mannheim)	27/26	12/11	4/12	20/28	*X*^2^ = 4.036 *P* = 0.258
Depression (BDI)	2.77 (3.01)	7.80 (7.96)	12.51 (10.07)	15.11 (12.03)	*F* = 15.220 *P* = < 0.001
Perceived stress scale	10.54 (5.20)	14.45 (6.90)	19.13 (5.10)	18.66 (8.76)	*F* = 11.874 *P* = < 0.001

Data are presented as mean (standard deviation) or number (%). Depression (*BDI* beck depression inventory) and Perceived stress scale. Chi-square test for independence was used for sex and site.

### Assessments of substance dependence

Classification of dependence for opioids, cannabis, and nicotine was based on the diagnostic guidelines of the International Classification of Diseases, 10th Revision (ICD-10), published by the World Health Organization [48]. A diagnosis of dependence required meeting at least three out of six ICD-10 criteria within the past 12 months. As applied in this study, ICD-10 dependence corresponds to severe substance use disorder as defined by DSM-5, which requires six or more of eleven criteria [49]. This equivalence is supported by the alignment of key diagnostic features across both systems, including craving, tolerance, and loss of control, and has been demonstrated empirically across alcohol, cannabis, and opioid use disorders [50]. In the current study, the mean number of ICD-10 criteria met in the primary substance group was 4.40 for OUD, 4.13 for CUD, and 4.00 for NUD, consistent with high diagnostic severity across all three groups. The term SUD is therefore used throughout this report as an umbrella term encompassing both classification frameworks. Diagnoses were assigned following short, structured interviews conducted by trained clinicians and researchers. Participants also reported lifetime history of substance-specific diagnoses to provide a comprehensive diagnostic profile (see supplementary Table S1). Previous additional SUD diagnoses beyond the primary substance were present in a proportion of participants: 16 of 46 CUD participants (34.8%), 13 of 15 OUD participants (86.7%), and 5 of 22 NUD participants (22.7%) had at least one previous additional SUD diagnosis. In the OUD group, lifetime diagnoses notably included cocaine dependence (31.3%), MDMA use (26.7%), amphetamine dependence (18.8%), and pregabalin use (18.8%), consistent with the clinical complexity typically observed in OST populations.

All opioid-dependent participants were receiving opioid substitution therapy (OST) at the time of scanning, using medications including Methadone, Polamidon, Buprenorphine, Morphine (Substitol), and Diamorphine. This group therefore reflected a pharmacologically stabilized OST sample, and the findings should not be generalized to individuals with active illicit opioid use.

To quantify nicotine dependence and account for its potential confounding effects on brain microstructure, participants completed the Fagerström Test for Nicotine Dependence (FTND; [51]). The FTND is a widely used six-item instrument designed to assess the physical dependence on nicotine. It captures aspects such as time to first cigarette after waking, number of cigarettes smoked daily, and difficulty refraining from smoking in restricted environments. The FTND yields a total score ranging from 0 to 10, with higher scores indicating greater nicotine dependence severity. For the current study, daily cigarette consumption (cigarettes per day) was used as the primary measure of nicotine exposure to align with cannabis and opioid severity measures.

Alcohol consumption was assessed as the average daily amount of alcohol intake expressed in grams per day, based on structured self-report interviews. This measure was used to characterize alcohol use patterns at the group level and was included as a covariate in all neuroimaging analysis. In addition, alcohol use severity was assessed using the Alcohol Use Disorders Identification Test (AUDIT; [52]). The AUDIT is a validated 10-item self-report instrument designed to capture hazardous and harmful alcohol consumption patterns. AUDIT scores were used descriptively to complement quantitative alcohol intake measures and to characterize risk-level drinking across groups.

In addition to the categorical SUD diagnoses, use patterns were quantified separately in terms of amount and frequency. For opioids use, the reported daily dosage of substitution medications (e.g., Methadone, Polamidon, Buprenorphine, Morphine/Substitol and Diamorphine) was converted to oral morphine-equivalent daily doses (MEDD) using multipliers primarily derived from clinical guidelines [53], with adjustments made in consultation with two clinical pharmacologists. Specifically, the following conversion multipliers were applied: 3.5 for Methadone, 7.0 for Polamidon, 33.0 for Buprenorphine (sublingual), 1.0 for Morphine (Substitol), and 1.5 for Diamorphine. These conversions reflect consensus-based estimates designed to standardize opioid load across pharmacologically distinct substances used in clinical practices.

For cannabis, the amount was recorded as the average number of joints (or joint-equivalent doses) consumed per day, based on participant self-report. Since precise tetrahydrocannabinol (THC) concentrations were not available, this measure represents a proxy for quantity that aligns with common epidemiological practices. For nicotine, the amount was quantified as the average number of cigarettes smoked per day, reflecting standard epidemiological measures. We specifically avoided reporting nicotine amount in grams, as this misrepresents actual consumption and absorption.

Frequency of substance use for each substance was assessed separately on an ordinal scale ranging from 1 to 6, reflecting patterns of use over the past year (1 = daily use; 2 = more than twice weekly; 3 = 1–2 times weekly; 4 = less than weekly; 5 = less than 1–2 times monthly; 6 = not frequently used). Due to pharmacokinetic differences and typical consumption patterns, frequency scores were interpreted only within each substance group and were not directly compared across groups.

All self-reported amount and frequency of substance use measures were collected through structured interviews conducted by trained research staff prior to scanning. Implausible values and outliers were identified and excluded following data quality checks.

### Behavioral assessment

All participants completed a standardized behavioral testing session prior to the MRI scan, lasting approximately 60 min. The behavioral battery was administered in a fixed order to all participants before scanning and consisted of three computerized tasks: the Kirby Delay Discounting, (KDDT) [54], the Stop-Signal Task, and the Go/No-Go task. Comprehensive instructions were provided at the beginning of each task, with administration via computer to ensure consistency in timing and presentation across both sites. For the current study only KDDT was analyzed. In this task, participants were presented with a series of 27 hypothetical choices between a smaller immediate monetary reward and a larger delayed reward, with imaginary reward amounts ranging from 11–85 euros and delays from 7 to 186 days. Each choice was unique, and participants indicated their preferred option in each case without receiving performance feedback. The task typically lasted 5 to 10 min. Delay discounting behavior was quantified by estimating each participant’s discount rate (k-value) using a hyperbolic discounting model, following established scoring procedures [54, 55]. Higher k-values reflected greater discounting, indicating a steeper preference for immediate over delayed rewards. Data quality was assessed by reviewing response patterns, and trials or participants with low completion rates (less than 90% completed trials) or evidence of random responding were excluded from analysis. Because k values were strongly positively skewed in the present sample (skewness = 1.98, Shapiro-Wilk *p* < 0.001), discount rates were natural log transformed prior to all parametric analyses. The KDDT has demonstrated strong reliability and validity in both clinical and non-clinical populations [56, 57].

### Image acquisition

Both study sites used identical acquisition protocols on a Siemens Prisma Fit scanner equipped with a 64-channel head-neck coil. Diffusion MRI data were acquired with anterior-to-posterior (AP) phase encoding direction and 2D echo-planar imaging (EPI) with TR/TE = 3200/63 ms, matrix = 110 × 110, 72 slices, 2 × 2 × 2 mm^3^ voxels, in-plane acceleration factor of 4, b = 1000/2000 s/mm², 60/120 diffusion-weighting directions (including 6 unweighted (b0) scans). To enable correction of susceptibility-induced distortions, an additional 6 b0 scans with reverse phase-encoding (posterior-to-anterior, PA) were acquired.

### Image processing and analysis

Data preprocessing was performed using established software pipelines and included denoising with MRtrix3 using (MP-PCA denoising), as well as correction for head motion, eddy current distortions, and susceptibility-induced artefacts using FSL (eddy and topup) [58–62]. Diffusion tensor imaging (DTI) metrics such as fractional anisotropy (FA) and mean diffusivity (MD) were derived from the b = 1000 s/mm² images. NODDI modelling was performed on the combined b = 1000 and b = 2000 s/mm² datasets using the Accelerated Microstructure Imaging via Convex Optimization (AMICO) framework [63]. This produced isotropic volume fraction (ISOVF), neurite density index (NDI) and orientation dispersion index (ODI) maps which were used for voxel-wise analyses. Whole-brain voxel-wise analyses of all diffusion metrics were conducted using tract-based spatial statistics (TBSS; as implemented in the FMRIB Software Library) [64].

### Tract-based spatial statistics (TBSS)

Voxel-wise analysis of FA, MD, ISOVF, NDI and ODI maps were carried out using TBSS. Individual FA maps were non-linearly registered to the FMRIB58_FA template in MNI152 standard space and subsequently aligned using FSL’s FNIRT tool [65]. A mean FA map was created from the standardized FA maps of all subjects and thinned to create the mean FA skeleton. The FA maps for each subject were then projected onto the mean WM skeletonized map and thresholded at FA > 0.20. All other diffusion metrics (i.e. MD, ISOVF, NDI, and ODI) were also warped onto the WM skeletonized map for subsequent analyses.

### Statistical analysis

**Fig. 1:**
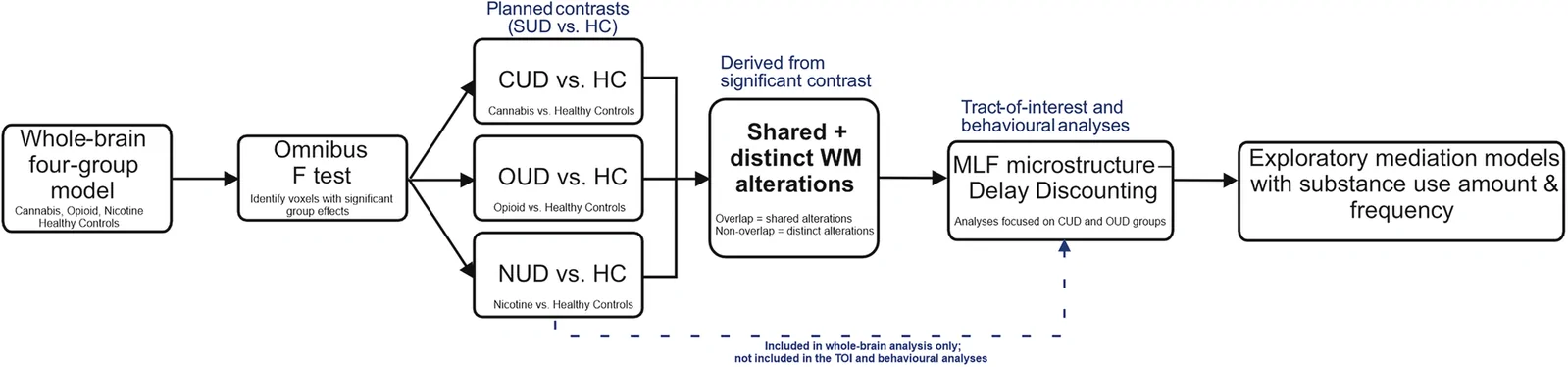
Flowchart of the hierarchical analytical strategy. Whole-brain TBSS analyses were conducted within a single four-group model including cannabis (CUD), opioid (OUD), nicotine (NUD), and healthy controls (HC). An omnibus F test was first used to identify voxels with significant group effects, followed by planned contrasts comparing each SUD group with HC. Shared and distinct WM alterations were derived from overlapping and non-overlapping significant regions across contrasts. Tract-of-interest (TOI) and behavior analyses focused on the CUD and OUD groups, examining associations between middle longitudinal fasciculus (MLF) microstructure and delay discounting. Exploratory mediation models were then performed to examine the influence of substance use severity (amount and frequency) on these associations. The NUD group was included in the whole-brain analyses to provide a reference pattern of nicotine-related alterations but was not included in the TOI-behavior analyses due to the absence of robust MLF involvement and limited behavioral relevance in this group.

Group comparisons of diffusion metrics were performed within a general linear model using non-parametric permutation testing with 10,000 permutations, implemented in FSL’s randomise tool [66]. Whole brain analyses were conducted within a single four group model including opioid, cannabis, nicotine, and healthy control participants. An omnibus F test was first used to identify voxels showing significant group effects across the model (see Fig. 1), with significance defined as *p* < 0.05, familywise error (FWE)-corrected. Planned post hoc contrasts were then used to compare each SUD group with healthy controls. For these three planned contrasts, results were additionally interpreted using a Bonferroni adjusted threshold of *p* < 0.017. Covariates included age, sex, site, and daily alcohol consumption (in grams per day), all of which were z-transformed prior to entry into the model. Threshold-Free Cluster Enhancement (TFCE) was applied to enhance cluster-wise inference. Based on the significant pairwise contrasts, overlapping regions of alteration were used to characterize shared WM abnormalities, whereas non-overlapping regions were used to identify distinct patterns of alteration across the cannabis, opioid, and nicotine groups.

Effect sizes were estimated from the Bonferroni-corrected post hoc TBSS results by extracting subject-level diffusion values from significant skeleton voxels within anatomically defined WM tracts from TractSeg [67]. Cohen’s d values were then computed for each tract and diffusion metric by comparing substance-dependent groups with healthy controls. Tracts were subsequently ranked according to absolute effect size to provide a descriptive comparison of WM effects across the substance-dependent groups and a quantitative complement to the voxelwise maps. Detailed tract-level rankings are presented in the Supplementary Results.

### MLF - delay discounting behavior relationship

**Fig. 2:**
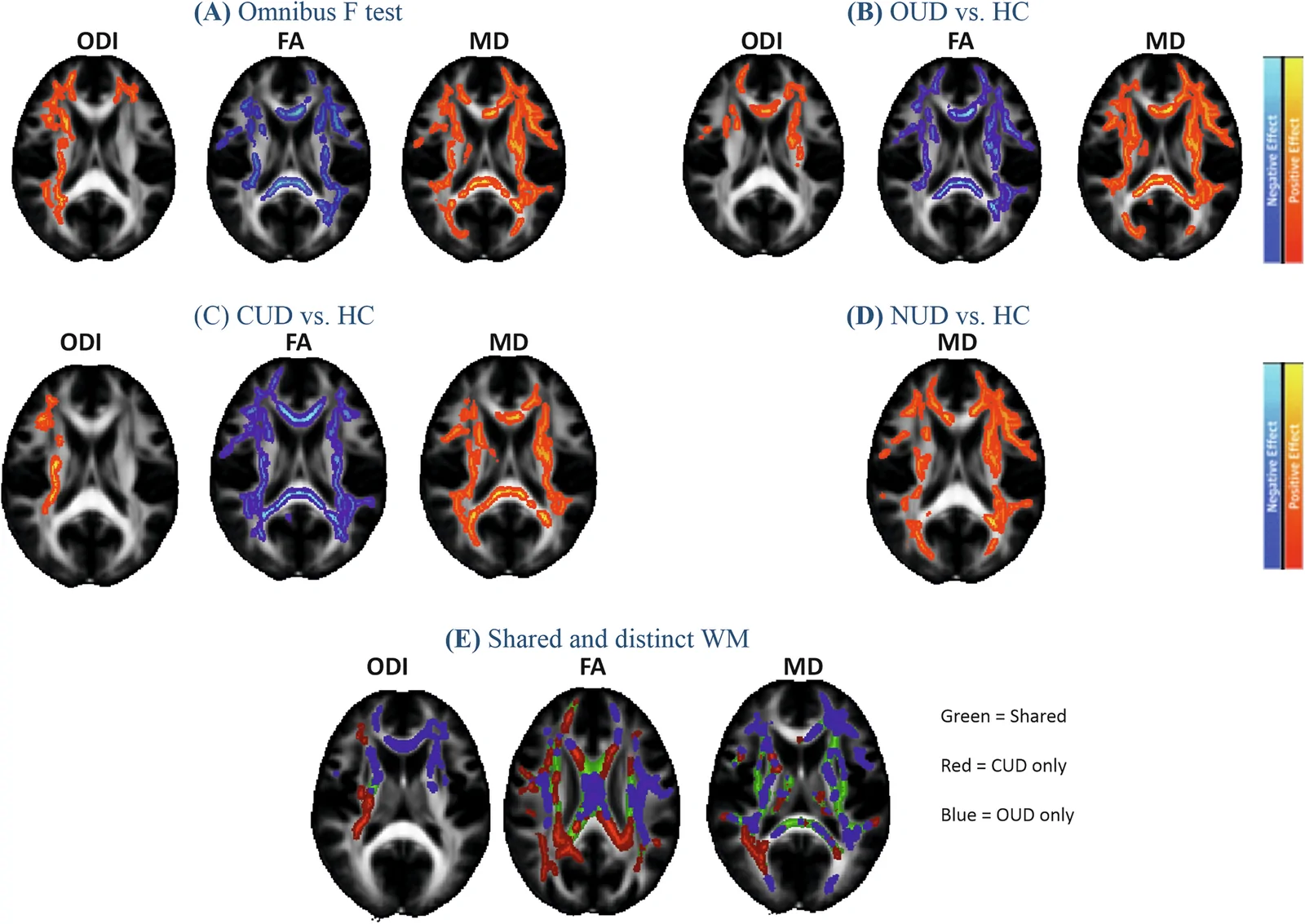
Whole-brain white matter microstructural alterations across SUD groups. Voxel-wise comparisons of ODI, FA, and MD were performed using tract-based spatial statistics within a single four-group model including OUD, CUD, NUD, and HC. **A** shows omnibus F test results. **B**–**D** show Bonferroni-corrected planned contrasts for each SUD group versus HC. Warm colors indicate higher ODI or MD in SUD relative to HC, whereas blue indicates lower FA in SUD relative to HC. **E** shows shared and distinct alterations derived from the significant OUD and CUD contrasts, with green indicating shared regions, red indicating CUD-distinct regions, and blue indicating OUD-distinct regions. Alterations were located primarily in association and limbic pathways, including the superior longitudinal fasciculus, middle longitudinal fasciculus, arcuate fasciculus, inferior longitudinal fasciculus, corpus callosum, anterior thalamic radiation, and internal capsule. NUD showed a more circumscribed pattern and was therefore not included in the shared and distinct overlap analysis. Axial slices are shown in MNI space (Z = 85 to 90) over the mean FA background.

Prior to analyzing brain-behavior relationships, ANOVA was performed to test for delay discounting differences across all groups (OUD, CUD, NUD, and HC). Subsequently, for the tract of interest-behavior analyses, we primarily focused on the OUD and CUD groups. This decision was guided by the whole-brain TBSS results, which indicated more prominent and widespread microstructural alterations in individuals with opioid and cannabis dependence compared to those with nicotine dependence (see Fig. 2). Moreover, the pharmacologically unstable steady state of nicotine use, combined with the high prevalence of nicotine co-use within the cannabis and opioid groups (see Table 2), pose methodological challenges for isolating distinct nicotine-specific brain-behavior relationships. For these reasons, nicotine use was treated as a covariate in all models, while the primary analyses were restricted to the OUD and CUD groups.

**Fig. 3:**
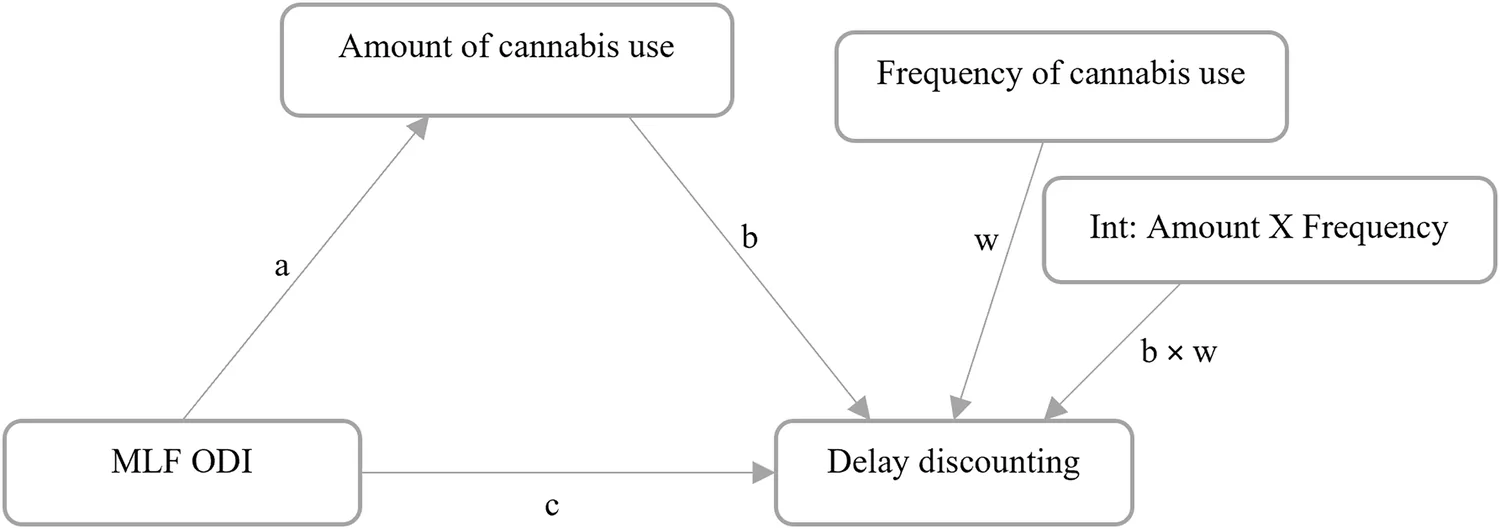
Exploratory moderated mediation model examining associations among MLF ODI, cannabis use amount, cannabis use frequency, and delay discounting in the CUD group. See summary table below for detailed report on each path.

White matter tracts across the entire brain were delineated using TractSeg [67], which automatically generated binary masks for 72 well-known tracts. We selected the left and right middle longitudinal fasciculus (MLF) and merged them to create a bilateral MLF mask. Mean FA and ODI values were extracted from the bilateral MLF, as these measures were supported by prominent representation in our TBSS results (see Fig. 2). Pearson’s partial correlations were computed between MLF values (FA and ODI) and delay discounting for OUD and CUD groups, controlling for age, sex, scanning site, daily alcohol consumption, and FTND scores. To account for multiple comparisons arising from testing two MLF measures (i.e. FA and ODI) against delay discounting, a Bonferroni threshold of *p* < 0.025 was used. To further examine the role of substance use severity in the observed associations, two exploratory mediation models were conducted using PROCESS (Model 14) in SPSS (version 29) with 5000 bootstrap samples. These analyses tested whether frequency and amount of cannabis or opioid use were associated with the relationship between MLF microstructure and delay discounting. In the primary model reported in the main text, substance use amount was specified as the mediator, with frequency moderating the mediator-outcome path, and MLF microstructure predicting delay discounting (see Fig. 3). In a supplementary model, MLF microstructure was tested as the mediator, capturing the possibility that substance use severity influenced delay discounting indirectly via alterations in WM microstructure. Bonferroni correction was applied across eight tests (two mediation model structures x two groups × two MLF metrics), yielding an adjusted significance threshold of *p* < 0.00625. Given the cross-sectional design of this study, these mediation analyses were treated as exploratory. No causal or temporal interpretation was inferred. Rather, they were used to examine statistical dependencies and to generate hypotheses for future longitudinal work.

## Results

### Clinical characteristics

Participant characteristics are summarized in Table 1. Compared to non-smoking healthy controls, all SUD groups reported elevated depressive symptoms that in most cases did not exceed the threshold for clinical depression, as assessed by the Beck Depression Inventory (BDI), with the highest mean scores observed in the cannabis and opioid groups (*F(3,136)* = 15.22, *p* < 0.001). Similarly, perceived stress, as measured by the Perceived Stress Scale (PSS), also differed significantly across groups (*F(3,136)* = 11.87, *p* < 0.001), with elevated scores particularly evident in OUD and CUD groups. These findings reflect a broader pattern of increased affective burden among opioids and cannabis groups.

Patterns of substance use differed significantly across SUD groups (see Table 2 for details). Daily cannabis consumption was highest in the CUD group (1.38 ± 0.96 joints/day), followed by the OUD group (0.81 ± 0.60), and lowest in the NUD group (0.37 ± 0.65), *F(2, 78)* = 10.00, *p* < 0.001. Post hoc comparisons indicated significantly higher cannabis use in the CUD group compared with the NUD group (*p* < 0.001); no other group comparisons reached significance. Nicotine consumption also differed significantly across groups, *F(2, 83)* = 10.15, *p* < 0.001, with the highest number of cigarettes per day reported in the NUD group (17.17 ± 7.58), followed by the OUD group (12.06 ± 7.19) and the CUD group (8.71 ± 7.38). Post hoc tests revealed significantly greater nicotine use in the NUD group compared with the CUD group (*p* < 0.001). Among individuals with opioid dependence, the average morphine-equivalent daily dose (MEDD) was 233.09 ± 219.05 mg/day. Although group differences in alcohol consumption reached overall significance, *F(3, 136)* = 2.76, *p* = 0.045, post hoc comparisons did not reveal any significant pairwise differences between groups after correction

In terms of substance use frequency, significant group differences were observed for cannabis use, *H(2)* = 14.60, *p* < 0.001. Post hoc Mann-Whitney U tests indicated that cannabis was used more frequently in the CUD group (mean rank = 27.16) compared to the OUD group (mean rank = 47.50; *p* < 0.001). No significant differences in frequency were found for nicotine or opioid use.

**Table 2:** Patterns of substance usage.

	Control *n* = 53	Nicotine dependence (NUD) *n* = 23	Cannabis dependence (CUD) *n* = 48	Opiate dependence (OUD) *n* = 16	Statistics	Post-Hoc
Alcohol consumption (g/day)	25.66 (21.04)	47.13 (50.07)	42.79 (42.61)	35.82 (29.12)	*F* = 2.758 *p* = 0.045	*n.s*.
AUDIT	3.26_39_ (3.08)	3.75_21_ (2.61)	5.86_39_ (5.64)	7.91_14_ (7.91)	*F*= 4.260 *p* = 0.007	*OUD > HC (p = .015)*
FTND (Nicotine Dependence)	1.53_17_ (2.21)	3.95_20_ (1.10)	3.10_40_ (1.36)	3.80_15_ (1.61)	*F*= 8.936 *p* < 0.001	*NUD > HC (p < 0.001)* *CUD > HC (p = 0.002)* *OUD> HC (p < 0.001)*
Past year amount consumed						
*Cannabisuse (joints/day)*	-	CU_18_ = 0.37 (0.65)	CU_48_ = 1.38 (0.96)	CU_15_ = 0.81 (0.60)	*F* = 10.000, *p* <0.001	*CUD > NUD (p < 0.001)*
*Nicotine use (cigarettes/day)*	-	NU_23_= 17.17 (7.58)	NU_47_ = 8.71 (7.38)	NU_16_ = 12.06 (7.19)	*F* = 10.148, *p* < 0.001	*NUD > CUD (p < 0.001)*
*Opioid substitution dose* *(MEDD, mg/day)*	-	-	-	OU_16_ = 233.09 (219.05)	-	-
Frequency of use (past year)	- - -	CU_18_ = 2.47 (2.44) NU_23_ = 1.13 (0.46) -	CU_48_ = 1.15 (0.36) NU_47_ = 1.21 (0.075) -	CU_15_ = 2.47 (1.41) NU_16_ = 1.19 (0.75) OU_16_ = 1.13 (0.50)	*H* = 14.60, *p* < 0.001 *H* = 0.25, *p* = 0.881 -	*CUD > OUD (p < 0.001)*

Data are presented as mean (standard deviation). For substance dependence groups, the number of users for each individual substance is additionally reported in subscript. Cannabis amount reflects average number of joints per day. Nicotine use is reported in cigarettes per day. Opioid substitution doses were collected from the OUD group and converted to morphine-equivalent daily doses (MEDD) using consensus-based multipliers: 3.5 for Methadone, 7.0 for Polamidon, 33.0 for Buprenorphine (sublingual), 1.0 for Morphine (Substitol), and 1.5 for Diamorphine [53]. Frequency of substance use was assessed using an ordinal scale: 1 = daily, 2 = more than twice per week, 3 = one to two times per week, 5 = less than one to two times per month, and 6 = not frequently consumed. *P*-values reported for alcohol consumption (g/day), FTND total score, cannabis use, and nicotine use are based on one-way between-groups ANOVA. Frequency of substance use was compared using the Kruskal-Wallis H test. Post hoc comparisons for ANOVA models were conducted using Bonferroni corrections.

*OU* opioid use, *CU* cannabis use, *NU* nicotine use, *FTND* Fagerström test for nicotine dependence, *AUDIT* alcohol use disorder identification test.

### Behavioral differences in delay discounting between SUD and HC groups

Significant group differences in delay discounting were observed using KDDT, which assesses the tendency to prefer smaller immediate over larger delayed rewards. Mean discount rates were highest in the opioid dependence group (M = 0.084, SD = 0.085), followed by the nicotine dependence group (M = 0.061, SD = 0.095), the cannabis dependence group (M = 0.052, SD = 0.063), and healthy controls (M = 0.045, SD = 0.046). Because discounting rates were strongly positively skewed, k values were natural log transformed before inferential testing. An ANOVA on ln(k) revealed a significant main effect of group on discounting rates, *F(3,132)* = 9.91, *p* < 001. Bonferroni-corrected post hoc comparisons showed that the OUD group exhibited significantly higher discount rates compared with HC (mean difference in ln(k) = 1.06, *p* < 0.001), reflecting stronger preference for immediate over delayed rewards in the OUD group. No other pairwise significant group differences were observed between the CUD, NUD and HC.

### Whole brain group comparison

Whole-brain voxel-wise TBSS analyses within the four-group model revealed significant omnibus group effects for FA, MD, and ODI after TFCE correction at p_FWE-corrected_ < 0.05 (see Fig. 2A). Omnibus effects were located predominantly in bilateral fronto-temporo-parietal and callosal WM regions. Planned contrasts comparing each SUD group with healthy controls were then examined to characterize the direction and spatial distribution of these effects. Compared to HC, the OUD group exhibited broadest pattern of WM alterations, characterized by reduced FA and increased MD and ODI across bilateral association and projection pathways. These effects were most apparent in fronto-temporo-parietal WM and extended into midline tracts, with involvement of regions consistent with the superior longitudinal fasciculus (SLF), middle longitudinal fasciculus (MLF), arcuate fasciculus (AF), anterior thalamic radiation (ATR), corpus callosum, and adjacent anterior projection pathways. The CUD group showed a similar but somewhat less extensive pattern, with bilateral FA reductions and increased MD and ODI across fronto-parietal, temporal, and limbic white matter regions, including the SLF, MLF, AF, corpus callosum, and cingulum bundle. In contrast, the NUD group demonstrated more localized pattern of alteration. Significant effects were observed primarily for MD, with clusters located in anterior and callosal WM regions. These NUD-related alterations fell largely within the broader spatial patterns observed in the OUD and CUD groups. Whole-brain voxel-wise comparisons of the additional NODDI metrics, NDI and ISOVF, showed no significant group differences surviving FWE correction in any contrast.

Direct overlap effects between the OUD and CUD groups revealed a substantial set of shared alterations (green, Fig. 2E), located primarily within fronto-temporo-parietal tracts (SLF, MLF, AF) and medial limbic pathways such as the cingulum. OUD-distinct alterations (blue) extended further into midline and projections structures such as the corpus callosum and ATR whereas CUD-distinct alterations (red) were more restricted to fronto-parietal and temporal association fibers. Shared and distinct maps were derived from binarized Bonferroni-adjusted FWE-corrected TBSS results, with categorical color coding used to depict spatial distribution; these visualizations do not convey the direction or magnitude of effects.

To further quantify the magnitude and distribution of WM effects across substances, tract-wise effect sizes were computed for each diffusion metric and substance group relative to healthy controls. Mean Cohen’s d values were extracted within significant TBSS skeleton voxels and summarized across anatomically defined WM tracts from TractSeg [67]. This analysis provides a ranked overview of tract-level effect sizes for cannabis, opioid, and nicotine dependence across FA, MD, and ODI. The full tract-wise effect size rankings and corresponding visualizations are presented in Supplementary Fig. S1.

### Association between MLF microstructure and delay discounting in cannabis and opioid dependent groups

Higher ODI in MLF, a key association tract known to connect temporal, parietal, and occipital regions with prefrontal areas involved in executive control, identified in our TBSS analyses, was significantly associated with increased delay discounting scores in the CUD group (r_(42)_ = 0.41, *p* = 0.011; Table 3). This finding shows that greater disorganization of MLF microstructure, reflected in higher ODI values, was associated with stronger preferences for immediate over delayed rewards. Delay discounting scores were also strongly associated with the amount of cannabis consumed, r_(42)_ = 0.69, *p* < 0.001. No significant associations were found between delay discounting and fractional anisotropy of the MLF (MLF FA), r_(42)_ = −0.24, *p* = 0.146, or with cannabis use frequency, r_(42)_ = 0.02, *p* = 0.898.

**Table 3:** Partial Correlations between MLF Microstructure, Delay Discounting, and Cannabis Use Severity in Cannabis dependence group.

Variable	1	2	3	4
1. DD	—			
2. MLF ODI	0.41*	—		
	**[0.118, 0.653]**			
3. MLF FA	−0.24	−0.51**	—	
	[−0.518, 0.056]	**[−0.723, −0.198]**		
4. Amount of cannabis use	0.69***	0.35*	−0.21	—
	**[0.342, 0.786]**	**[0.045, 0.608]**	[−0.487, 0.146]	
5. Frequency of cannabis use	0.02	0.21	−0.10	0.18
	[−0.353, 0.450]	[−0.134, 0.510]	[−0.403, 0.289]	[−0.175, 0.487]

*N* = 48. Values are Pearson’s partial correlations controlling for age, sex, site, daily alcohol consumption, and FTND scores. Values in brackets represent 95% bootstrapped confidence intervals (5000 samples).

*MLF* middle longitudinal fasciculus, *FA* fractional anisotropy, *ODI* orientation dispersion index, *DD* delay discounting (higher = greater impulsivity).

* p < 0.05. ** *p* < 0.01. *** *p* < 0.001.

In the OUD group, no significant associations were observed between delay discounting and bilateral MLF ODI r_(7)_ = 0.43, *p* = 0.254; 95% CI [−0.967, 1.000], or bilateral MLF FA r_(7)_ = −0.27, *p* = 0.176; 95% CI [−0.608, 0.103]. Delay discounting was also not significantly associated with opioid MEDD, r_(7)_ = −0.33, *p* = 0.097; 95% CI [−0.688, 0.080].

### Primary moderated mediation model: associations between MLF ODI, cannabis use severity, and delay discounting

Given the significant associations between MLF ODI and delay discounting in the CUD group, we used an exploratory moderated mediation model (PROCESS Model 14; see Fig. 3) to examine associations among MLF ODI, cannabis use severity, and delay discounting. In this model, MLF ODI was entered as the independent variable (X), delay discounting (Kirby k-value) as the dependent variable (Y), cannabis use amount as the mediator (M), and cannabis use frequency as the moderator (W) of the M → Y path. Overall, the model was significant and explained 50.7% of the variance in delay discounting (*F(4, 41)* = 10.54, *p* < 0.001, R² = 0.507).

As shown in Table 4, MLF ODI significantly predicted the amount of cannabis use (a-path: 16.925, *p* = 0.024). Although the main effect of the amount of cannabis use on delay discounting (b-path) was not significant overall (b = −0.025, *p* = 0.308), the interaction between cannabis use amount and frequency was significant (b = 0.054, *p* = 0.016). Conditional effects (Table 5) revealed that cannabis use amount was positively associated with delay discounting at both  frequency levels. However, the conditional indirect effect of MLF ODI on delayed discounting through cannabis amount was significant only at the daily use level (b = 0.396, 95% CI [0.023, 1.186]). No significant conditional indirect effect was observed for moderate frequency users (more than twice per week; 95% CI [−0.159, 3.201]). These findings should be interpreted as associative rather than directional, given the cross-sectional design.

**Table 4:** Summary of the exploratory moderated mediation model.

Path / Effect	Coefficient (b)	SE	t	p	95% CI [LL UL]
a-path: MLF ODI → Amount of cannabis use	16.925	7.226	2.342	**0.024**	[**2.363 31.488]**
b-path: Amount → Delay discounting (direct effect)	−0.025	0.025	−1.036	0.308	[−0.071 0.024]
w-path: Frequency → Delay discounting	0.031	0.020	1.584	0.123	[−0.095 0.026]
Interaction (b × w)	**0.054**	0.021	2.581	**0.016**	[**0.010 0.098**]
Total R²	—	—	—	—	**0.507 (*p*** < **0.001)**

Model summary for the moderated mediation analysis. Bootstrap results are shown in this table. *N* = 48. Bootstrap sample size = 5000.

*LL* lower limit of 95% confidence interval, *UL* upper limit of 95% confidence interval. Only two levels of cannabis use frequency were represented in this group: daily use and more than twice per week (but not daily).

**Table 5:** Conditional Effects of Cannabis Use Amount on Delay Discounting at Different Frequency Levels.

Frequency of Use	Effect	SE	t	p	95% CI [LL UL]
Daily (1)	0.034	0.008	4.305	**0.000**	**[0.018 0.050]**
2x/Week (2)	0.088	0.021	4.301	**0.000**	**[0.047 0.130]**
Conditional Indirect Effects (Bootstrapped) MLF ODI → Amount of cannabis use → Delay discounting					
Daily (1)	0.396	0.304	—	—	**[0.023 1.186]**
2x/Week (2)	1.150	1.017	—	—	[-0.159 3.201]

Bootstrapped Results for the Conditional Effects of Cannabis Use Amount on Delay Discounting at Different Frequency Levels Note: Only two levels of cannabis use frequency were represented in this group: daily use and more than twice per week (but not daily).

### Supplementary exploratory reverse model: associations between cannabis use severity, MLF ODI, and delay discounting

To address uncertainty regarding directionality, a supplementary exploratory reverse model was also tested. In this model, cannabis use severity was entered as the independent variable (X), MLF ODI as the mediator (M), and delay discounting as the outcome (Y). The overall model was significant, *F(4, 41)* = 8.504, *p* < 0.001, with R² = 0.452. Consistent with the primary moderated-mediation analysis, greater cannabis use amount significantly associated with higher MLF ODI (b = 0.007, *p* = 0.024), and higher MLF ODI was positively associated with delay discounting (b = 0.040, *p* = 0.028).

However, the indirect effect of cannabis use amount on delay discounting through MLF ODI was not significant, as the 95% bootstrap confidence interval included zero (Boot 95% CI [−0.008, 0.009]). Thus, although this pattern is consistent with an association among cannabis use severity, MLF ODI, and delay discounting, the indirect effect was not statistically supported in our sample and should be interpreted as exploratory.

## Discussion

This study revealed widespread white matter (WM) microstructural alterations across individuals with substance use disorders (SUD) compared to non-smoking healthy controls (HC). Using tract‐based spatial statistics (TBSS) with both neurite orientation dispersion and density imaging (NODDI) and diffusion tensor imaging (DTI), opioid- and Cannabis-dependent groups showed more extensive WM microstructural alterations within fronto-temporo-parietal and callosal WM pathways. Within these findings, the middle longitudinal fasciculus (MLF) emerged as a tract of interest, given its role in connecting temporal and parietal regions to prefrontal networks implicated in cognitive control and reward valuation. In the CUD group, high orientation dispersion index (ODI) in the MLF was associated with steeper delay discounting. Exploratory moderated mediation analyses further suggested that this association was statistically related to cannabis use amount, with a significant conditional indirect effects observed only among daily users. In contrast, although the OUD group showed more widespread WM alterations overall, no significant association between MLF microstructure and delay discounting was observed.

### Shared and distinct white matter alterations across SUDs

Our TBSS analysis revealed significant omnibus group effects for FA, MD and ODI across the four-group model, with effects predominantly located in bilateral fronto-temporo-parietal and callosal WM regions including the corpus callosum, superior longitudinal fasciculus (SLF), inferior longitudinal fasciculus (ILF), anterior thalamic radiation (ATR), internal capsule, and MLF— regions supporting executive function, reward processing, and cognitive control. These findings echo prior work showing broad microstructural compromise in SUD populations [17, 68] and align with the view that chronic substance exposure exerts neurotoxic or neurodevelopmental impacts on fronto‐limbic and associative networks [21].

Planned contrasts then showed broad alterations in OUD, a similar but less extensive pattern in CUD, and a more circumscribed pattern in NUD. The OUD and CUD groups demonstrated extensive bilateral FA reductions and increase MD/ODI within fronto-temporo-parietal pathways. These findings extend prior work showing opioid-related WM alterations in limbic-thalamocortical tracts [24, 25, 69, 70] and cannabis‐related deficits in the arcuate/SLF and corpus callosum [23, 71]. In the present data, overlap between OUD and CUD was observed primarily within bilateral association pathways and adjacent callosal regions, whereas non-overlapping effects showed a broader extension into midline and projection regions in OUD and a more lateralized association-fiber pattern in CUD. These results support the presence of both shared and distinct WM alterations across substance groups, while also indicating substantial overlap in their spatial distribution.

In contrast, the NUD group exhibited a more focal pattern of alteration, with significant effects observed primarily for MD in anterior and callosal WM regions. These findings are consistent with previous DTI studies that reported relatively circumscribed fronto‐limbic changes in nicotine dependence [30], likely reflecting differences in underlying mechanisms of action or a broader clinical characteristics in nicotine dependance [21]. Notably, individuals in the NUD group showed overall lower levels of psychiatric burden compared with the OUD and CUD groups (see Table 1), which may also contribute to the more restricted pattern of WM alterations observed.

To further quantify the relative magnitude of WM alterations across substance groups and diffusion metrics, tract-wise effect sizes were computed for each dependent group relative to HC. Mean Cohen’s d values were extracted within significant TBSS skeleton voxels and summarized across anatomically defined WM tracts, providing a ranked overview of tract-level effects across FA, MD, and ODI. These tract-wise effect size distributions presented in Supplementary Fig. S1, demonstrated that long range fronto-temporo-parietal association pathways consistently ranked among the most affected tracts across diffusion metrics and substance groups. Taken together, these results indicate both shared WM alterations across substances and distinct profiles that may relate to differences in pharmacology, chronic exposure, or clinical burden [72–76]. The fronto-temporo-parietal tract, which includes the MLF, consistent involvement across shared maps underscores its potential role as a transdiagnostic marker of cognitive and affective dysfunction in SUD. The predominance of MD effects further suggests that these alterations may reflect more global tissue properties, such as changes in extracellular space or glial-related processes, which have been shown to extend across white and gray matter compartments in substance-related pathology [77].

### The middle longitudinal fasciculus and delay discounting

Among the WM tracts identified, the MLF emerged as a tract of particular interest given its anatomical role in connecting the superior temporal gyrus to superior parietal lobule, and terminates in dorsolateral prefrontal regions [44, 78, 79]. This pathway is thought to support integration of sensory, language, and higher‐order executive functions, including temporal‐reward valuation and inhibitory control [80, 81]. Alterations in the MLF have been associated to increased preference for immediate rewards, commonly measured using delay discounting [42].

In our CUD subgroup, higher ODI in the bilateral MLF was significantly associated with steeper delay discounting (r = 0.41, *p* = 0.011), even after controlling for nicotine use. This association reflects variability within the cannabis group rather than differences between diagnostic groups. ODI reflects the dispersion of neurite orientations within a voxel and is sensitive to local fiber geometry and crossing fibers [36, 37], and therefore does not map directly onto a specific biological process. Accordingly, the observed association between higher MLF ODI and steeper delay discounting is best interpreted as altered microstructural signal within a behaviorally relevant tract, rather than tract‑specific fiber disorganization. This pattern is consistent with the view that variation in microstructural properties within the MLF may be related to impulsive decision making in CUD, although the underlying biological basis remains uncertain [42, 82, 83].

In contrast, FA in the MLF did not significantly relate to discounting (r = −0.24, *p* = 0.146), suggesting the sensitivity of NODDI-derived indices such as ODI for capturing microstructural features related to cognitive-behavioral outcomes [34, 38].

Interestingly, in the OUD group, despite the presence of widespread WM alterations, neither ODI nor FA in the MLF was significantly associated with delay discounting or MEDD. The absence of a significant MLF-delayed discounting association in OUD should be interpreted cautiously, given the small sample size and clinically heterogeneous OUD sample, which limited statistical power to detect moderate effects. This finding should therefore not be taken as evidence of a true neurobiological difference from CUD [84]. Opioid-related WM alterations may involve alternative circuits, such as thalamocortical or limbic striatal pathways, although this interpretation remains tentative [85].

### Associations between cannabis use severity, MLF ODI, and delay discounting?

Consistent with the dual-process and dose-response models of addiction [86], our exploratory moderated mediation analyses indicated that the association between MLF ODI and delay discounting in CUD was statistically related to cannabis use severity. Specifically, higher MLF ODI was associated with greater cannabis consumption, and cannabis use amount was positively associated with delay discounting at both observed frequency levels; however, the conditional indirect effect through cannabis amount was significant only among daily  users (Table 5). This frequency-dependent pattern suggests that altered MLF microstructure may be associated with heavier cannabis use, but that its association with impulsive choice is most evident among daily users.

This interpretation is consistent with evidence that heavier cannabis exposure is associated with greater neuroanatomical alteration and cognitive difficulties [87]. For example, Filbey and Yezhuvath [88], demonstrated that heavy, daily cannabis users exhibit greater fronto‐parietal functional connectivity disruptions and poorer inhibitory control than occasional users. Similarly, Jacobus et al. [89], reported that heavy cannabis use in adolescence is associated with lower FA in association tracts. Thus, our exploratory moderated mediation findings are consistent with a conceptual model in which MLF microstructure, consumption dose, and use frequency are statistically related to impulsive decision making in CUD. Nevertheless, these findings do not support a specific causal or temporal sequence in the present cross-sectional design.

### Exploratory reverse model: associations between cannabis use severity, MLF ODI, and delay discounting

In a supplementary exploratory reverse model, cannabis use severity was specified as the predictor and MLF ODI as the mediator. Although cannabis use amount was associated with higher ODI, and higher ODI was associated with steeper delay discounting, the indirect effect was not statistically significant after bootstrapping and Bonferroni correction. Relative to the primary model, this reverse model provided less support for an indirect association. Taken together, however, both models should be interpreted as associative rather than directional.

### Differential profiles in OUD versus CUD groups

Despite showing widespread WM alterations, the OUD group did not exhibit a significant MLF-delay discounting association. This finding should be interpreted cautiously, given the small and clinically heterogeneous OUD sample, which may have limited power to detect tract-behavior relationships. In addition, the OUD group showed the highest levels of perceived stress and state anxiety symptoms, both of which are known correlates of impulsive choice [8, 90]. This affective burden may have obscured tract-specific associations with delay discounting. Opioid-related WM abnormalities are also more consistently reported in thalamocortical, limbic, and reward-related pathways, including the cingulum and internal capsule, with MLF involvement likely to be secondary or nonspecific [25, 70, 91]. Accordingly, impulsive choice in OUD may be linked more closely to circuits beyond the MLF. Overall, our findings suggest that while both CUD and OUD groups showed shared WM vulnerabilities, an association between MLF microstructure and delay discounting was detected only in CUD in the present sample.

### Limitations

Several limitations temper the interpretation of our findings. First, the cross-sectional design precludes causal inference. Although moderated mediation analyses identified potential pathways linking MLF disorganization, cannabis use severity, and impulsivity, it remains unclear whether WM abnormalities precede heavier cannabis use and steeper discounting, or whether chronic exposure contributes to these changes. Longitudinal and interventional studies together with well-controlled animal experiments (see e.g. De Santis, Bach, et al. [28]) are needed to address causality and directionality. Second, subgroup sample sizes were modest, particularly for nicotine (*n* = 23) and opioid (*n* = 16) groups, which limited statistical power and generalizability. Replication in larger, multisite samples would strengthen robustness and allow examination of moderators such as sex or age of onset. Third, while our analyses focused on the MLF based on TBSS results and prior literature [44, 79], other WM tracts involved in executive control and reward processing may likely contribute to impulsivity across SUDs. Future multimodal work should investigate network-level contributions beyond tract-specific effects. Fourth, although prior substance exposure histories, particularly in the OUD group, may have contributed to residual variance in WM microstructure, current comorbid SUD was an exclusion criterion in the current study. Our findings should therefore be interpreted in the context of previous rather than concurrent polysubstance use. Future work should employ analytic strategies such as latent profile analysis to better disentangle the effects of prior polysubstance use. Lastly, although site was included as a covariate in all voxel-wise analyses, significant site differences in global FA and MD among healthy controls indicate residual between site variance that should be considered when interpreting these metrics.

## Conclusion

This study provides convergent evidence that SUDs are associated with widespread WM microstructural alterations, most prominently in opioid- and cannabis-dependent individuals. Both groups showed abnormalities in fronto-temporo-parietal and callosal pathways, captured by traditional DTI and advanced NODDI metrics, with both shared and distinct spatial patterns. In the CUD group, greater orientation dispersion in the MLF was associated with steeper delay discounting, and this association was statistically related to cannabis consumption patterns in exploratory analyses. In contrast, although OUD group also exhibited WM abnormalities, only CUD showed a significant association between MLF microstructure and impulsive choice in the present sample. Our findings support the relevance of MLF microstructure to delay discounting in cannabis dependence and underscore the importance of distinguishing shared and distinct WM alterations when characterizing the neurobiology of addiction. Given the cross-sectional design, the brain-behavior and mediation findings should be interpreted as associative and exploratory.

## Supplementary information

Supplementary information

## Data Availability

The data are not publicly available due to privacy and ethical restrictions.
