# Supplementary material for: White matter signatures of addiction diverge across opioids, cannabis and nicotine and predict impulsive choice in cannabis dependence
Source: Transl Psychiatry. 2026 Oct 9;16:548. doi: 10.1038/s41398-026-04491-8 (PMC13652822; doi:10.1038/s41398-026-04491-8)
Supplement: Supplementary file 1 [file 41398_2026_4491_MOESM1_ESM.docx]

**SUPPORTING INFORMATION**

**White matter signatures of addiction diverge across opioids, cannabis and nicotine and predict impulsive choice in cannabis dependence.**

Richard O. Nkrumah^1,2^ ,Lea Wetzel^2,3^ ,Justin Böhmer^4,5^ ,Katharina Eidenmueller^2,3^ , Patrick Bach^2,3^, Silvia De Santis^6^, Henrik Walter^4^, Wolfgang H. Sommer^2,3,7,8,*^ and Gabriele Ende^1,2,*^

^1^ Department of Neuroimaging, Central Institute of Mental Health, Medical Faculty Mannheim, Heidelberg University, J5, 68159 Mannheim, Germany

^2^ German Center for Mental Health (DZPG), partner site Mannheim-Heidelberg-Ulm, Mannheim, Germany

^3^ Department of Addictive Behavior and Addiction Medicine, Central Institute of Mental Health, Medical Faculty Mannheim, Heidelberg University, J5, 68159 Mannheim, Germany

^4^ Department of Psychiatry and Psychotherapy CCM, Charité - Universitätsmedizin Berlin, Corporate Member of Freie Universität Berlin, Humboldt-Universität zu Berlin, and Berlin Institute of Health, Berlin, Germany

^5^ Institute of Medical Psychology, Charité - Universitätsmedizin Berlin, Corporate Member of Freie Universität Berlin, Humboldt-Universität zu Berlin, and Berlin Institute of Health, Berlin, Germany

^6^ Spanish National Research Council Instituto de Neurociencias, CSIC/Universidad Miguel Hernandez, Alicante, Spain

^7^ Department of Psychopharmacology, Central Institute of Mental Health, Medical Faculty Mannheim, Heidelberg University, J5, 68159 Mannheim, Germany

^8^ Bethany Hospital for Psychiatry, Psychosomatics and Psychotherapy, 17489 Greifswald, Germany

^*^ These authors contributed equally to this work

**Supplementary Information**

White matter signatures of addiction diverge across opioids, cannabis and nicotine and predict impulsive choice in cannabis dependence.

The Supplementary Information provides additional extended information supporting the main manuscript. This includes detailed diagnostic characteristics and comorbidity profiles across substance use disorder groups, as well as tract wise effect size distributions derived from the

|  | | Control  n=53 | Nicotine dependence (NUD  n=23 | Opioid dependence (OUD)  n=16 | Cannabis dependence (CUD)  n=48 |
| --- | --- | --- | --- | --- | --- |
| Current | Alcohol diagnosis, % (n) | 82.6 (19) | 69.8 (37) | 62.5 (10) | 66.7 (32) |
|  | Cannabis diagnosis, % (n) | 13.0 (3) | 0.0 (0) | 68.8 (11) | 97.9 (47) |
|  | Nicotine diagnosis, % (n) | 87.0 (20) | 9.4 (5) | 100.0 (16) | 87.5 (42) |
|  | Opioid diagnosis, % (n) | 0.0 (0) | 0.0 (0) | 50.0 (8) | 0.0 (0) |
| Lifetime | Alcohol diagnosis, % (n) | 90.5 (19) | 84.1 (37) | 90.0 (9) | 90.3 (28) |
|  | Cannabis diagnosis, % (n) | 78.3 (18) | 39.6 (21) | 100.0 (9) | 100.0 (27) |
|  | Nicotine diagnosis, % (n) | 94.4 (17) | 28.8 (15) | 100.0 (7) | 96.4 (27) |
|  | Opioid diagnosis, % (n) | 17.4 (4) | 0.0 (0) | 100.0 (15) | 16.7 (8) |
| ICD-10 | Alcohol criteria, mean (SD) | 0.70 (1.40) | 0.08 (0.28) | 1.47 (1.85) | 0.62 (1.17) |
|  | Cannabis criteria, mean (SD) | 0.84 (1.46) | 0.03 (0.16) | 2.47 (1.46) | 4.12 (1.18) |
|  | Nicotine criteria, mean (SD) | 4.00 (0.85) | 0.07 (0.25) | 2.88 (1.26) | 2.45 (1.54) |
|  | Opioid criteria, mean (SD) | 0.19 (0.54) | 0.00 (0.00) | 4.40 (1.59) | 0.12 (0.45) |

Table S 1. Diagnostic characteristics and comorbidity profile across substance use disorder groups.

*Note. This table summarizes current and lifetime substance use diagnoses and ICD-10 dependence criteria across opioid, cannabis, nicotine dependence groups and healthy controls. Values represent percentage of participants meeting diagnostic criteria or mean and standard deviation of ICD-10 criteria counts. Diagnoses were derived from structured clinical interviews based on ICD-10 guidelines. This table complements Table 2 by providing detailed diagnostic profiles and comorbidity information beyond substance use amount and frequency.*

Figure S 1. Tract-wise effect size distributions across substance use disorder groups and diffusion metrics. Panels display ranked Cohen’s d values computed within significant TBSS skeleton voxels for cannabis, opioid, and nicotine dependence relative to healthy controls. Rows correspond to substance groups and columns to diffusion metrics. Positive values indicate higher diffusion measures in the substance group relative to controls, whereas negative values indicate lower values. Tracts were included if at least 50 significant skeleton voxels were present. Effect sizes are shown for visualization and comparison purposes and do not replace voxel-wise inference and may be upwardly biased because they are derived from significant TBSS voxels.
